# Neuropsychological aspects of digital distraction: a randomized controlled experimental study

**DOI:** 10.3389/fpsyg.2026.1873941

**Published:** 2026-08-12

**Authors:** Robert Krause

**Affiliations:** Department of Educational and School Psychology, Constantine the Philosopher University, Nitra, Slovakia

**Keywords:** cognitive interference, digital health, digital distraction, neuropsychology, social media

## Abstract

This randomized controlled experimental study examined the impact of intermittent digital distraction on cognitive executive functions within simulated social and work environments. The primary objective was to evaluate potential cognitive interference induced by audiovisual stimuli across behavioral (error rate), neurophysiological (spectral EEG parameters), and subjective (perceived workload via NASA-TLX) dimensions. The research sample comprised 87 adult participants (students, managers, and employees), randomly assigned to an experimental group ( n=45 ) exposed to standardized audiovisual distractions and a control group ( n=42 ). The experimental protocol utilized a battery of psychodiagnostic tasks assessing working memory (Dual N-back), inhibitory control (Stroop task), and selective attention (d2-R), synchronized with continuous 7-channel EEG recording via the LSL protocol. Data were analyzed using Generalized Linear Mixed Models (GLMM), ANCOVA, and Spearman partial correlations. Behavioral analysis revealed no statistically significant main effect of digital distraction on overall error rates in the Dual N-back and Stroop tasks. A potential interaction between group and stimulus congruency in the Stroop task was observed (*p* = 0.043); however, given the non-significant main interaction (*p* = 0.285), this trend requires cautious interpretation. Neurophysiological analysis identified severe correlation between fm-Theta and Alpha bands (*r* > 0.98), limiting the interpretation of their unique contributions. Subjective perceived workload significantly correlated with error rates exclusively during working memory tasks. In conclusion, under the current experimental conditions, intermittent audiovisual distraction did not result in a statistically significant degradation of primary task accuracy, suggesting that participants maintained behavioral performance over short time intervals.

## Introduction

In the current digital paradigm, an unprecedented level of connectivity facilitates information access but simultaneously subjects the human brain to chronic cognitive challenges. Digital technologies and notification systems exploit attentional mechanisms, competing with the executive functions of the prefrontal cortex (PFC) Haber et al. (2022). This state of attentional fragmentation and high cognitive load may result in a phenomenon known as “Cognitive Outflow” or “Brain Drain” (Ward et al., 2017), characterized by a depletion of available mental capacity due to external digital factors. Executive functions (EF) represent a complex set of cognitive processes necessary for goal-oriented behavior, primarily including inhibition, working memory, and cognitive flexibility (Ardila, 2008).

In a digital context, the integrity of EF can be compromised as exogenous stimuli (notifications) compete with endogenous control (the target task). During the processing of conflicting stimuli, the anterior cingulate cortex (ACC) acts as a cognitive conflict detector (Carter et al., 1998). According to the Miller and Cohen (2001) model, once a discrepancy between the target state and a distraction is detected, executive control mechanisms are engaged to suppress the disturbance. This process is accompanied by spectral fluctuations in EEG frequency bands, such as frontal midline Theta (fm-Theta) and Alpha rhythms (Klimesch, 2012).

According to the Miyake and Friedman (2012) model, executive functions share a common factor while exhibiting specific operations. Inhibition serves as a key mechanism; its failure under digital distraction may cascade into impairments in working memory Orban et al. (2015). Furthermore, switching between tasks generates switching costs and “attention residue” (Leroy, 2009)—a state where neural processing of a distractor continues after returning to the primary task. Ward et al. (2017) demonstrated that the presence of digital devices can reduce available cognitive capacity, as inhibitory control is continuously allocated to suppress the urge to check notifications. While chronic multitasking has been associated with structural variations in cortical regions (Loh and Kanai, 2014), experimental acute laboratory exposures allow us to observe functional changes in real-time performance and electrophysiological markers.

The suppression of a distractor via the rIFG-STN pathway (right inferior frontal gyrus – subthalamic nucleus) is a metabolically demanding process (Aron et al., 2014). When the brain is exposed to intermittent (unpredictable) stimuli, it undergoes constant “restarts” of executive processes, leading to metabolic depletion and cognitive exhaustion Aagaard (2015). Objective analysis of digital interference relies on three key neurophysiological markers:

1. fmTheta (4–8 Hz): An indicator of conflict detection and cognitive load.
2. Alpha Band (8–12 Hz): Associated with sensory gating; its desynchronization indicates a weakening of filtration mechanisms (Klimesch, 2012).
3. P300 Amplitude (ERP): Reflects the degree of resource allocation in working memory. A reduced amplitude in the presence of a distractor provides direct evidence of cognitive outflow.

Chronic exposure to digital interference also results in structural changes Arnsten (2009). Loh and Kanai (2014) identified a reduction in gray matter density in the ACC among individuals with high levels of media multitasking. This finding suggests that digitalization does not only alter behavior; it leads to a neuroplastic reconfiguration of the brain’s executive architecture.

### Research problem

The scientific problem of our work is to clarify the causal relationship between cognitive interference and the effectiveness of executive processes. We are interested in the extent to which acoustic and visual notifications modify neurophysiological brain activity and whether this continuous fragmentation of attention indicates a measurable increase in cognitive error rates and subjective load, resulting in significant degradation of performance in executive-demanding test batteries.

### Research focus

The main aim of the work is therefore to examine the impact of digital interference on executive functions and attentional focus in participants through neuropsychological analysis and a cross-subject experiment. The partial aim is to verify the existence of the phenomenon of cognitive drain (Ward et al., 2017) in a work and social context and to quantify the difference in the level of inhibitory control in participants exposed to cognitive interference compared to the control group in an environment without the presence of digital stimulants. The partial aim is also to analyze the change in the bioelectrical activity of the brain in real time during cognitive load with the presence of a distractor and to monitor the relationship between objectively measured performance and subjective perception of mental effort.

### Research aim and research questions

Based on the introduction, it follows that cognitive and digital interference is not just a form of external noise, but on the contrary, it is a process that significantly reorganizes the functioning of executive functions. This entire mechanism is influenced by cognitive outflow (Ward et al., 2017), but also by the metacognitive strategy of cognitive interpretation (Storm and Stone, 2015). In addition, the capacity of working memory (Cowan, 2001), which is limited, but also the energy demand associated with the detection of cognitive conflict by the anterior cingulate cortex, also enters the whole process. In addition, the entire mechanism of cognitive interference is also regulated at the biochemical level due to inhibition by the hyperdirect rIFG-STN pathway and is also influenced by the level of reward anticipation (Beer et al., 2006; Xue et al., 2008). Based on the assumption that long-term exposure to digital technologies and the distractions they generate can also lead to changes in gray matter density (Loh and Kanai, 2014), we will also look at objective verification of these changes as part of our experimental design.

Reflecting the theoretical background and the aim of the experiment, we decided to formulate the following research questions:

*RQ1*: To what extent does audiovisual digital distraction increase the error rate (ER%) in tasks focused on working memory (Dual N-back) and inhibitory control (Stroop test)?
*RQ2*: Is there a significant difference in the overall concentration performance (CP) in the d2-R test between the experimental group exposed to distractions and the control group?
*RQ3*: What is the relationship between the error rate in cognitive tests and subjectively felt mental effort (NASA-TLX)?
*RQ4*: Is the increased error rate in tests manifested by a specific neurophysiological profile (e.g., alteration of the fm-Theta and Alpha bands)?
*RQ5*: To what extent does subjectively perceived cognitive load (NASA-TLX) correlate with objective neurophysiological parameters (Alpha and Theta bands) in the studied groups?

## Research methodology

### General background

The scientific research is conceived as a complex experimental design carried out in a controlled environment of a psychological laboratory with the aim of objectifying the impact of intermittent digital distraction on executive functions in a heterogeneous sample of students and employees. The main methodological pillar is the integration of a proprietary software application that synchronizes behavioral psychodiagnostic tasks (Dual N-back, Stroop test) with continuous neurophysiological recording using a 7-channel EEG system Brain Products X.on. The experimental procedure simulates common digital interference in real time through standardized audiovisual notifications with an intensity of 60 dB, which are inserted into the process at pseudo-random intervals. The accuracy of data collection is ensured by the LSL protocol, which allows for rigorous segmentation of neurophysiological markers, namely spectral analysis of fm-Theta and Alpha rhythms, as well as analysis of event-related potentials (P300 amplitude). Objectively measured parameters of executive error rate and reaction times are supplemented in the final phase with subjective scaling of mental load using the NASA-TLX questionnaire and measurement of residual capacity of selective attention using the d2-R test.

### Sample

The research sample (*n* = 87) consisted of male (*n* = 12) and female (*n* = 18) psychology teachers aged 19–24 (AM = 21.4; SD = 1.2) from the University of Constantine the Philosopher in Nitra, who were selected for our research by purposive sampling. Our research also included male (*n* = 16) and female (*n* = 13) managers from various companies aged 28–54 (AM = 41.2; SD = 7.8) who participated in the research based on an online call. Ordinary employees from various companies (*n* = 28) aged 24–58 (AM = 36.7; SD = 9.4) also participated in the research. We randomly divided the research participants into an experimental (*n* = 45) and a control (*n* = 42) group by drawing lots. The study took place in the psychological laboratory at the Department of Educational and School Psychology, Constantine the Philosopher University in Nitra, Slovakia. Data collection was carried out over a period of 4 months, from October 2024 to March 2025. The laboratory environment was sound-attenuated, temperature-controlled ( $21^{\circ }C-22^{\circ }C$ ), and maintained under constant artificial lighting to minimize external sensory noise.

### Instruments and procedures

The experimental protocol consisted of a battery of five methods ordered by cognitive demand and measurement goal, with an emphasis on objectifying working memory and inhibition processes. The duration of the measurement for one participant was 30 min.

#### Dual N-back

This multimodal task is considered one of the most demanding paradigms for measuring working memory (Jaeggi et al., 2008). It requires simultaneous processing of auditory and visual stimuli, while activating the fronto-parietal network, especially the dlPFC and ACC (Kerns et al., 2004). The method tests the limited capacity of working memory slots (Cowan, 2001) through the processes of updating, inhibition and attention switching.

#### NASA TLX (task load index)

A multidimensional rating scale (Hart, 2006) serves to objectify the subjective perception of cognitive load. It tracks six dimensions: mental, physical and time demands, performance, effort and frustration. The design allows for a comparison of objective performance with the degree of conscious effort expended on suppressing distractions via the rIFG-STN pathway.

#### Stroop test

A neuropsychological method (Stroop, 1935) aimed at measuring cognitive interference arising from a conflict between automated reading and conscious color naming. The test serves as a detector of the effectiveness of inhibitory control, while monitoring the interference score and the prolongation of reaction time to incongruent stimuli.

#### d2-R attention test

A performance test (Brickenkamp et al., 2010) measures selective attention and visual discrimination under time pressure. The inclusion of the “pencil-paper” method at the end of the battery allows for the quantification of residual mental fatigue and stability of concentration (CP index) after previous exposure to intermittent digital distractors.

#### EEG headset X.on (brain products)

A 7-channel wireless system using active sensor technology to eliminate electromagnetic noise. The device allows millisecond synchronization with the stimulation software via the LSL protocol, which is crucial for the analysis of event-related potentials and spectral analysis of waves (fm-Theta, Alpha) in the prefrontal regions of the brain.

### Data analysis

Data analyses were performed in the statistical software environment R (version 4.4.3), using several specialized packages. The dplyr, tidyr, and readr packages were used for data manipulation and transformation. The lme4 and lmerTest packages were used for mixed models, sandwich and lmtest for calculating robust standard errors and testing models, and robustbase for robust regression. The effsize package was used for calculating effect sizes.

### Research results

Inferential analysis of RQ1 examined the effect of audio-visual distraction on error rates (ER%) in the Dual N-back and Stroop tasks. Given the non-normal distribution of error rates (Shapiro–Wilk; Stroop: *W* = 0.486, *p* < 0.001; Dual N-back: *W* = 0.866, *p* < 0.001) and the presence of a floor effect in the Stroop task (skewness = 3.34), a generalized linear mixed model with logit transformation was applied. The model did not reveal a statistically significant interaction between group and task type (*b* = −0.41, SE = 0.38, z = −1.07, *p* = 0.285, OR = 0.66), indicating that the effect of distraction did not differ between the working memory and inhibition domains. The main effect of task type was significant (*b* = −1.71, SE = 0.28, z = −6.20, p < 0.001, OR = 0.18), with the error rate in the Dual N-back task (*M* = 23.9%) being significantly higher than in the Stroop task (*M* = 6.8%). The main effect of group was not statistically significant (*b* = 0.22, SE = 0.29, *z* = 0.76, *p* = 0.448, OR = 1.24), which is also confirmed by the descriptive data:

*Dual N-back*: control group (*M* = 23.69, SD = 20.50) vs. experimental group (*M* = 24.14, SD = 20.42).
*Stroop test*: control group (*M* = 7.50, SD = 15.97) vs. experimental group (*M* = 6.22, SD = 12.14).

Analysis of covariates (age: *p* = 0.880; gender: *p* = 0.305; education: *p* = 0.403) showed no systematic effect on error rates.

The results do not provide empirical support for the assumption that intermittent digital distraction increases error rates in working memory or inhibitory control tasks.

Research Question 2 (RQ2) examined whether a significant difference exists in total concentration performance (CP) on the d2-R test between the experimental group (exposed to distractions) and the control group. All assumptions for the Analysis of Covariance (ANCOVA) were met, including the normality of distribution (Shapiro–Wilk: *W* = 0.978, *p* = 0.148), normality of residuals (*W* = 0.981, *p* = 0.255), and homogeneity of variances [Levene’s test: *F*(1,84) = 0.003, *p* = 0.954]. Homogeneity of regression slopes was confirmed, and multicollinearity was negligible (VIF < 1.10 for all predictors). Descriptive statistics indicated a numerically higher average performance in the experimental group (*M* = 120.57, SD = 43.61) compared to the control group (*M* = 113.29, SD = 48.53), though high inter-individual variability was observed in both samples. The ANCOVA revealed no statistically significant main effect of the group (*F* = 1.77) = 0.47, *p* = 0.496, partial *n*^2^=0.006. The regression coefficient for the difference between groups remained non-significant [*b* = −6.91, SE = 10.11, *t*(77) = −0.68, *p* = 0.496, 95% CI (−27.04, 13.22)] after controlling for all covariates. Among the included covariates, age (*p* = 0.461), gender (*p* = 0.944), education (*p* = 0.675), and accuracy in the Stroop task (*p* = 0.129) showed no significant effect. In contrast, accuracy in the Dual N-back task was a significant predictor of concentration performance [*F*(1,77) = 4.64, *p* = 0.034, partial *n*^2^ = 0.57, *b* = 54.70, SE = 25.40, *t* (77) = 2.15]. This indicates that higher working memory accuracy was associated with higher concentration performance in the d2-R test. The overall model explained 13.5% of the variance in concentration performance (*R*^2^ = 0.135, adjusted *R*^2^ = 0.045). In conclusion, the results do not suggest that intermittent audio-visual distraction leads to a decrease in concentration performance on the d2-R test. Instead, performance differences appear to be driven by individual differences in working memory capacity rather than the experimental manipulation itself.

Research Question 3 investigated the relationship between cognitive error rates and subjective mental workload measured by the NASA-TLX subscales (Effort, Frustration, Mental Demand). Due to the non-normal distribution of error variables, Spearman’s correlation (*ρ*) was employed. To control for Type I error across nine correlations, the Holm–Bonferroni correction was applied.

*Stroop task*: No significant relationships were found after correction. Although the correlation between error rate and frustration was initially *ρ* = 0.29 (*p* = 0.008), it became non-significant after Holm’s adjustment (p_Holm_ = 0.064). Relationships with effort (*ρ* = 0.21, p_Holm_ = 0.260) and mental demand (*ρ* = 0.18, p_Holm_ = 0.376) also remained non-significant.
*Dual N-back task*: Moderate positive correlations were identified across all subscales. Higher error rates significantly correlated with higher effort (*ρ* = 0.34, p_Holm_ = 0.010), frustration (*ρ* = 0.41, p_Holm_ = 0.006) and mental demand (*ρ* = 0.37, p_Holm_ = 0.008). These findings indicate that subjective workload is a robust indicator of objective performance specifically in working memory tasks.
*d2-R test*: Correlations between error rates and subjective load were weak and non-significant for effort (*ρ* = 0.09), frustration (*ρ* = 0.15), and mental demand (*ρ* = 0.12). In summary, the link between subjective perception of load and objective error rates was task-specific. While the Dual N-back task showed a clear alignment between high cognitive cost and perceived strain, the Stroop and d2-R tasks did not exhibit such convergence, suggesting that participants’ awareness of their own performance may be more accurate in high-demand working memory paradigms.

To investigate whether increased error rates are associated with a specific neurophysiological profile (RQ4), two separate hierarchical linear regressions were conducted for the Stroop and Dual N-back tasks. The models were based on 76 participants, with the experimental group included as a covariate in the first step and log-transformed EEG indicators (frontal theta and parietal alpha) added in the second step.

#### Key methodological constraints

- Non-normality: Significant deviations from normal distribution were observed in error rates and EEG variables (*p* < 0.001), requiring cautious interpretation of the inferential conclusions.
- Severe Multicollinearity: A critical methodological issue emerged as frontal theta and parietal alpha were extremely highly correlated (*r* > 0.98 in both tasks). High Variance Inflation Factors (VIF > 40) indicate that the unique contribution of each frequency band cannot be reliably separated; therefore, the models only assess the EEG block as a collective predictor.

#### Results by task

- Stroop Task: The initial model was non-significant (*R*^2^ = 0.003, *p* = 0.649). Adding EEG variables provided no meaningful improvement to the variance explained (*R*^2^ = 0.003, *p* = 0.908), and the final model remained non-significant (*F*(3.72) = 0.13, *p* = 0.941). Neither group, theta, nor alpha power served as significant predictors of error rates.
- Dual N-back Task: Similar to the Stroop task, the first step was non-significant. Incorporating EEG power increased the variance explained to *R*^2^ = 0.041, but this change was not statistically significant (*F*(2.72) = 1.52, *p* = 0.225). Although a slight positive trend for theta and a negative trend for alpha were observed, neither reached statistical significance.

The hierarchical regression analyses provide no evidence that increased error rates in either task are significantly associated with specific theta or alpha activity. In both cases, neurophysiological indicators failed to add predictive value beyond the group covariate. Furthermore, the extreme correlation between the EEG bands suggests that any potential relationship with performance would likely be tied to a shared neural component rather than specific frequency band activity.

We validated our RQ5 in which we investigated to what extent subjectively felt cognitive load (NASA-TLX) correlates with objective neurophysiological parameters (Alpha and Theta bands) in the studied groups?

To examine the relationship between subjectively felt cognitive load and neurophysiological parameters, we used Spearman partial correlations, while also controlling for the group variable. We conducted the analysis separately for the Stroop and Dual N-back tasks and included the relationships between the total NASA-TLX score and log-transformed measures of frontal theta and parietal alpha activity.

The results of the analysis in the Stroop task showed no significant relationship between subjectively felt workload and EEG parameters, which indicates the absence of a consistent link between the internal experience of effort and neurophysiological indicators in this domain. In contrast, in the Dual N-back task, a statistically significant negative relationship was identified between subjective cognitive workload and parietal alpha activity, indicating that an increase in perceived mental effort was directly correlated with a decrease in alpha oscillations. Overall, it can be stated that the correlation between subjective effort and neurophysiological activity was selectively manifested only in tasks focused on working memory, while this relationship was not confirmed in other cognitive tasks.

## Discussion

The interpretation of the findings in the framework of the presented scientific research brings a comprehensive view of the dynamics between digital interference and the executive apparatus of the human brain. When analyzing the first research question (RQ1), which examined the error rate in tasks focused on working memory and inhibitory control under the influence of audio-visual distraction, the results indicate a remarkable ability of short-term adaptive resilience of the executive system. Although Ward et al. (2017) postulate that the suppression of irrelevant stimuli itself consumes a critical amount of energy and leads to cognitive drain, our participants were able to maintain performance accuracy even under experimental conditions. This phenomenon can be explained through the lens of Kurzban et al.’s (2013) cognitive effort model, according to which subjective psychological experience and mobilization of internal resources enable the suppression of distractors without immediate performance degradation. In practice, it was shown that participants perceived digital notifications as unwanted noise and consciously increased cognitive input to eliminate the error rate, which is also confirmed by the observed “floor effect” in the Stroop task. However, the higher error rate in the Dual N-back test compared to the Stroop test (*p* < 0.001) indicates that the working memory updating processes are more executively demanding than pure motor or cognitive inhibition, although in neither case did distraction cause system collapse. Reflecting the findings of Aron et al. (2014), the absence of prolongation of reaction times suggests that the hyperdirect rIFG-STN pathway was not paralyzed by the presence of digital stimuli, which indicates the stability of cognitive control across demographic covariates such as education or gender, thereby partially correcting the assumptions of Knox and Wolohan (2014) about the influence of cognitive reserve on short-term resilience.

In the second research question (RQ2), which monitored the stability of concentration in the d2-R test after exposure to distractions, it was shown that cumulative cognitive fatigue did not leave a significant trace in the subsequent selective attention ability (*p* = 0.496). This result is scientifically intriguing, especially in the context of Cowan’s model (2011), which assumes that the suppression of distractors occupies slots in working memory, which theoretically should lead to subsequent exhaustion. However, our data suggest the presence of cognitive plasticity and compensation (Ding et al., 2025), where the increased alertness (arousal) induced by distraction persists and paradoxically stabilizes subsequent performance. The key finding of VO2 remains that the strongest predictor of concentration in the d2-R test was accuracy in the Dual N-back test (*p* = 0.034), confirming that individual working memory capacity and prefrontal circuit stability are more dominant for performance than external stimuli. From a neuropsychological perspective, this implies that individuals with a more robust central executive are able to switch more effectively between demanding cognitive operations and routine administrative control, with this stability acting as a protective factor against residual fatigue from digital interference.

The third research question (RQ3) focused on the relationship between error rate and subjectively felt mental effort (NASA-TLX), with the results yielding a differentiated picture depending on the type of task. A significant correlation between error rate and subjective load (effort *ρ* = 0.34; frustration *ρ* = 0.41) was confirmed exclusively in the Dual N-back test. We explain this finding through metacognitive processing (Menon and D'Esposito, 2022), where participants were clearly aware of moments of failure and loss of continuity during demanding information updating, which led to an increase in perceived frustration. On the contrary, this correlation was absent in the Stroop and d2-R tests, suggesting that inhibition processes controlled by more autonomous rIFG-STN pathways (Aron et al., 2014) may not be perceived by consciousness as enormously demanding unless they lead to massive interference. Thus, digital distraction appears to increase the “cost of performance” especially where there is a direct competition for slots in working memory (Ward et al., 2017), while during routine administrative tasks individuals may not be aware of the latent weakening of their attention, which may be a risk factor in a real work environment.

The fourth research question (RQ4) investigated the existence of a specific neurophysiological profile associated with increased error rates. The analysis encountered methodological limitations in the form of extreme multicollinearity between theta and alpha activity (r > 0.98), suggesting that in our experimental setting these bands represented a common neurophysiological substrate of cortical excitation. Nevertheless, the results yielded a crucial finding about the stability of the alpha rhythm in the experimental group (*p* = 0.002). Instead of the expected fragmentation of attention, intermittent distraction led to a significant increase in the stability of alpha activity, which we interpret as a neurophysiological “shield” or state of hyper-focus (Ashinoff and Abu-Akel, 2021). This sensory gating mechanism (Klimesch, 2012) allowed participants to suppress digital stimuli as irrelevant noise, thereby proactively protecting the attention devoted to the primary task (Braver, 2012; Anderson and Levy, 2009). The stabilization of parietal neuronal networks thus serves as a reactive tool for selective resource targeting in the short term.

The last research question (RQ5) analyzed the correlation between subjective workload and neurophysiological parameters (fm-Theta and Alpha). While no relationship was found in the Stroop task, a significant negative correlation was identified between perceived effort and parietal alpha activity in the Dual N-back task (*ρ* = −0.31). Lower levels of alpha activity at high perceived workload indicate that the central executive had to make a massive effort to maintain the goals in memory (Klimesch, 2012). However, an interesting finding is the absence of an increase in fm-Theta activity in the experimental group, which contradicts the models of Langner et al. (2018) and Guo et al. (2018). This fact suggests that participants habituated to the distraction very quickly and their ACC did not evaluate it as a priority cognitive conflict (Goldin-Meadow et al., 2001). Paradoxically, at higher perceived effort, fm-Theta activity even decreased (*r* = −0.31), indicating that at maximum concentration the brain suppressed the monitoring of the external environment in favor of the primary goal. This neuroplastic correspondence between perceived effort and suppression of conflict detection suggests that the executive system is capable of effective adaptation even to numerous digital distractions, as long as the goal is perceived as a priority, although the long-term metabolic and psychological consequences of such hypervigilance require further scientific investigation.

## Conclusions and implications

The main scientific contribution of the presented work is the elucidation of the mechanisms by which the human brain adapts to digital interference, with the key finding being the ability of the executive system to perform short-term cognitive compensation. It has been shown that with sufficient goal prioritization and high cognitive task difficulty, the central executive can effectively eliminate the negative impact of distractors at the level of primary performance, allowing participants to maintain accuracy even under conditions of intermittent distraction. From a neuropsychological perspective, we have objectified this protection process through increased stability of the alpha rhythm in parietal areas, which we interpret as a state of proactive control and the creation of a specific “neurophysiological shield.” Although this mechanism successfully minimizes error rates, the results indicate that it is associated with an increase in the energy intensity of information processing and increased subjectively felt frustration, especially in the domain of working memory. In contrast to the original assumptions, the research did not demonstrate a moderating effect of formal education on resistance to distraction, which suggests that in the digital environment, the current capacity of executive functions and individual neurophysiological plasticity are more crucial than the accumulated cognitive reserve.

The findings of the work have direct implications for the practice of management, education and diagnostics. Organizations are recommended to introduce systemic “no-distraction” zones that eliminate the need for energy-intensive suppression of digital noise, thereby preventing premature cognitive exhaustion and burnout. At the pedagogical level, it seems necessary to focus on training working memory, which serves as a key protective factor enabling students to more effectively gate irrelevant stimuli. The results also warn against the phenomenon of “digital neutrality,” when the brain stops detecting conflict during frequent multitasking, which can lead to long-term cognitive instability.

From a clinical and psychological diagnostic perspective, it is critical that short-term performance can remain normal despite distraction due to compensatory mechanisms; therefore, assessment of competence (e.g., in drivers) should take into account, in addition to error rate, the degree of perceived exertion and the length of exposure. Overall, the work underlines that although the human brain is able to resist digital distractors for the short term without loss of accuracy, conscious suppression of notifications during executively demanding tasks remains a neurophysiological necessity for maintaining mental health and optimal performance in a digitalized society.

## Data Availability

The raw data supporting the conclusions of this article will be made available by the authors, without undue reservation.
